# Perioperative Nutrition and Surgical Outcomes in Patients Receiving Glucagon-like Peptide-1 Receptor Agonists: A Scoping Review

**DOI:** 10.3390/medicina62081434

**Published:** 2026-07-23

**Authors:** Tegbir Singh Sandha, Ofir Ron, Warren M. Rozen, Ishith Seth, Roberto Cuomo

**Affiliations:** 1 Department of Plastic and Reconstructive Surgery, Peninsula Health, Frankston, VIC 3199, Australiaishithseth1@gmail.com (I.S.);; 2 Faculty of Medicine and Surgery, Peninsula Clinical School, Monash University, 2 Hastings Road, Frankston, VIC 3199, Australia; 3 Plastic Surgery Unit, Department of Medicine, Surgery and Neuroscience, University of Siena, Banchi di Sotto, 55, 53100 Siena, SI, Italy

**Keywords:** GLP-1 receptor agonists, perioperative nutrition, surgical outcomes, body composition, sarcopenia, wound healing

## Abstract

*Background and Objectives*: Glucagon-like peptide-1 receptor agonists (GLP-1RAs) are increasingly prescribed for obesity and type 2 diabetes mellitus. While these agents provide substantial metabolic benefits, concerns have emerged regarding their effects on nutritional status and potential implications for perioperative outcomes. *Materials and Methods*: A scoping review was conducted in accordance with PRISMA-ScR guidelines. A structured literature search was conducted from 2 May 2026 to 8 June 2026 examining GLP-1RA therapy, nutritional status, body composition and surgical outcomes. Eligible studies included investigations of postoperative outcomes associated with GLP-1RA use, nutritional and body composition effects of GLP-1RAs, associations between nutritional abnormalities and surgical outcomes, and perioperative nutritional optimisation strategies. Data were extracted and synthesised narratively. *Results*: Twenty studies met the inclusion criteria. Four major themes were identified: (1) nutritional and body composition changes associated with GLP-1RA therapy, (2) effects of malnutrition, sarcopenia, myosteatosis and micronutrient deficiencies on surgical outcomes, (3) postoperative outcomes associated with GLP-1RA use, and (4) perioperative nutritional assessment and optimisation strategies. GLP-1RA therapy was consistently associated with reduced energy intake, inadequate protein intake, micronutrient deficiencies and loss of lean body mass. These abnormalities overlap with recognised risk factors for adverse postoperative outcomes. Despite this, clinical studies generally reported neutral or favourable postoperative outcomes among GLP-1RA users, particularly in arthroplasty and hand surgery populations, although increased wound-healing complications were reported in selected plastic surgery cohorts. *Conclusions*: Current evidence suggests that GLP-1RAs do not consistently worsen postoperative outcomes; however, their use is associated with nutritional and body composition changes that may influence perioperative recovery. Nutritional assessment and optimisation may therefore represent an important component of perioperative care in patients receiving GLP-1RA therapy.

## 1. Introduction

Glucagon-like peptide-1 receptor agonists (GLP-1RAs) have emerged as an increasingly important therapeutic class for the management of obesity and type 2 diabetes mellitus. Their use has expanded rapidly in recent years due to their effectiveness in promoting substantial and sustained weight loss, improving glycaemic control, and reducing cardiovascular risk [1,2,3]. As the prevalence of obesity continues to rise worldwide, a growing proportion of patients presenting for elective and reconstructive surgical procedures are receiving GLP-1RA therapy.

Despite increasing clinical interest, the existing literature remains fragmented across surgical specialties and study designs. Current evidence encompasses investigations of postoperative outcomes in patients receiving GLP-1RA therapy, studies examining the nutritional and body composition effects of these agents, and research evaluating the consequences of malnutrition, sarcopenia and micronutrient deficiencies on surgical recovery [4,5,6,7,8]. However, these evidence streams have largely been investigated independently, and no previous review has synthesised their interrelationship within a perioperative context. Existing reviews have primarily focused on perioperative medication management, delayed gastric emptying or procedure-specific postoperative outcomes, whereas the nutritional consequences of GLP-1RA therapy have generally been examined separately from surgical outcomes. Integrating these distinct but related areas of evidence may provide a broader understanding of how GLP-1RA therapy could influence perioperative risk and help identify priorities for future research and nutritional optimisation strategies.

The aim of this scoping review was therefore to map and synthesise the available evidence regarding the relationship between GLP-1RA therapy, nutritional status and surgical outcomes. Specifically, this review explores: (1) nutritional and body composition changes associated with GLP-1RA therapy, including protein inadequacy, micronutrient deficiencies and loss of lean body mass; (2) the impact of malnutrition, sarcopenia, myosteatosis and micronutrient deficiencies on postoperative outcomes; (3) postoperative outcomes associated with perioperative GLP-1RA use; and (4) current approaches to perioperative nutritional assessment and optimisation aimed at mitigating nutritional risk and improving surgical recovery. By integrating evidence relating to GLP-1RA-associated nutritional and body composition changes, established nutritional risk factors for poor postoperative outcomes, and direct clinical surgical evidence and perioperative nutritional optimisation strategies, this review proposes a conceptual framework to inform future perioperative research and clinical practice.

## 2. Methodology

### 2.1. Study Design

A scoping review was undertaken to map and synthesise the existing literature examining the relationship between GLP-1RAs, nutritional status, body composition, and perioperative surgical outcomes. A scoping review methodology was selected due to the emerging nature of the evidence base and the anticipated heterogeneity in study populations, interventions, outcome measures, and study designs. The review was conducted in accordance with the Preferred Reporting Items for Systematic Reviews and Meta-Analyses Extension for Scoping Reviews (PRISMA-ScR) framework. A review protocol was not prospectively registered.

### 2.2. Search Strategy

A structured literature search was conducted using the PubMed database between 2 May and 8 June 2026. PubMed was selected because it provides comprehensive coverage of biomedical and clinical literature and was considered appropriate for the exploratory objectives of this scoping review. The search strategy was developed following an initial exploration of the literature to identify relevant terminology and indexing. Search terms combined concepts relating to GLP-1RAs, obesity pharmacotherapy, nutrition, malnutrition, protein deficiency, sarcopenia, body composition, wound healing, perioperative care, surgical complications, and postoperative outcomes using a combination of Medical Subject Headings (MeSH) and free-text keywords where appropriate. Boolean operators (AND/OR) were used to combine search concepts and maximise retrieval of relevant studies. The complete search strategy is provided in the Supplementary Materials. Search results were imported into Zotero (version 9.0.4) reference management software for organisation and duplicate removal. Reference lists of included studies and relevant review articles were manually screened to identify additional eligible studies not captured through the electronic database search. The literature search, title and abstract screening, full-text assessment, and data extraction were conducted by a single reviewer.

### 2.3. Eligibility Criteria

Studies were eligible for inclusion if they met one or more of the following criteria:

1. Investigated the association between GLP-1RA use and postoperative or wound-healing outcomes.
2. Evaluated the effects of GLP-1RA on nutritional status, dietary intake, body composition, lean mass, sarcopenia, or micronutrient status.
3. Examined the relationship between malnutrition, sarcopenia, myosteatosis, protein deficiency, or micronutrient deficiencies and surgical outcomes.
4. Evaluated perioperative nutritional assessment, nutritional optimisation strategies, prehabilitation, or nutrition-focused interventions relevant to surgical patients.
5. Included adult human populations undergoing surgery or receiving GLP-1RA therapy.
6. Included preclinical mechanistic studies where they provided direct evidence relevant to the biological mechanisms underlying wound healing, nutritional status, body composition, or surgical outcomes associated with GLP-1RAs.
7. Were published in English and available in full-text form.

Studies were excluded if they:

1. Included paediatric populations.
2. Were not relevant to perioperative nutrition, body composition, wound healing, or surgical outcomes.
3. Focused exclusively on the glycaemic or weight-loss effects of GLP-1RAs without reporting nutritional, body composition, or surgical outcome measures.
4. Were conference abstracts, editorials, correspondence articles, expert opinions, or duplicate publications.
5. Were animal or basic-science studies that did not provide mechanistic evidence directly relevant to perioperative nutrition, body composition, wound healing, or surgical outcomes.

### 2.4. Study Selection

**Figure 1:**
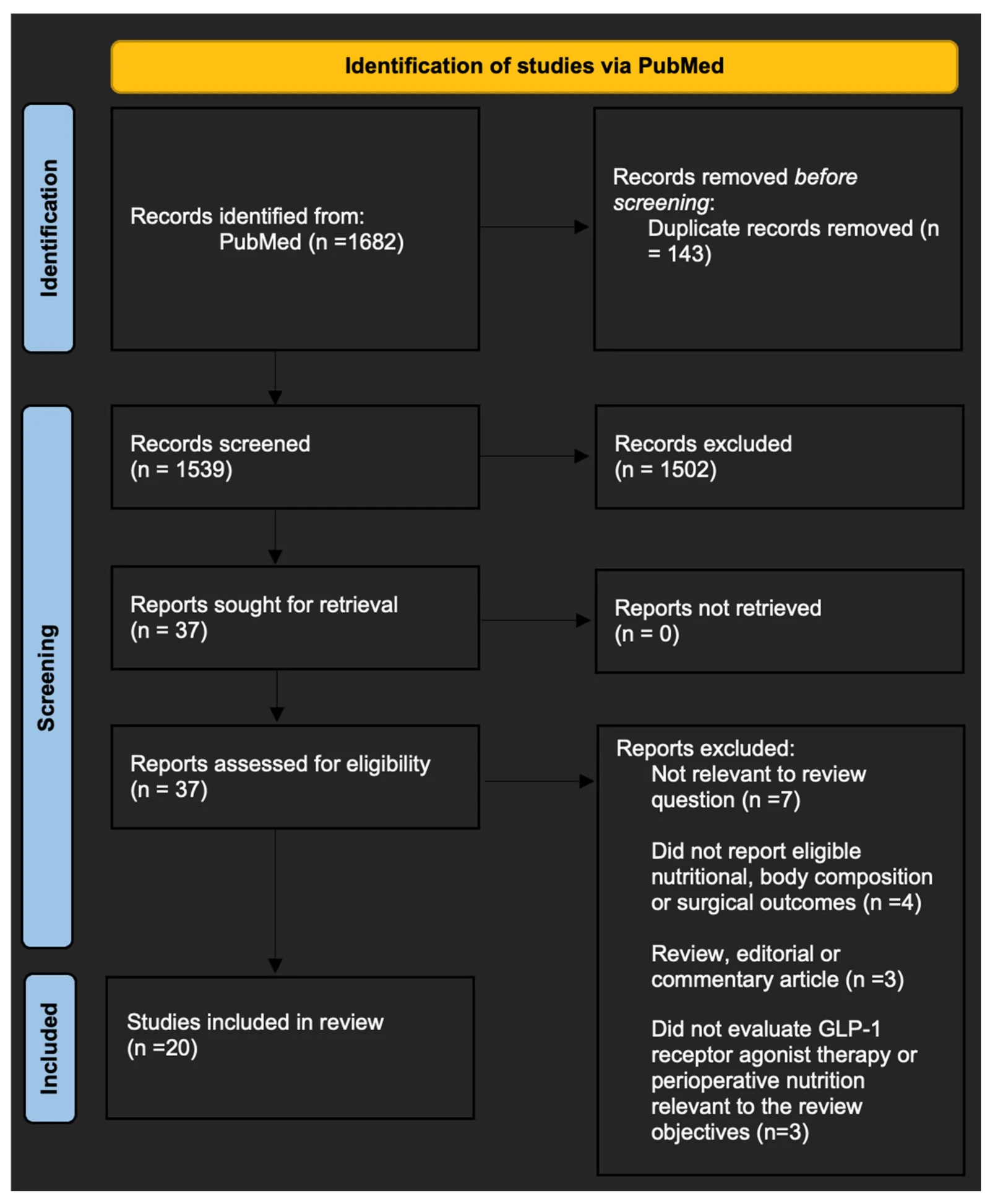
PRISMA flow diagram demonstrating the identification, screening, eligibility assessment and inclusion of studies examining the relationship between GLP-1RA therapy, nutritional status and perioperative outcomes.

All records retrieved from the literature search were imported into Zotero reference management software, where duplicate records were identified and removed. Titles and abstracts were screened for relevance to the review objective by a single reviewer. Potentially eligible studies subsequently underwent full-text assessment against the predefined inclusion and exclusion criteria. Reasons for exclusion were documented where appropriate, and studies meeting all eligibility criteria were included in the final synthesis. Reference lists of included studies and relevant reviews were screened manually to identify any additional eligible publications. The study selection process is summarised in Figure 1 (PRISMA flow diagram).

### 2.5. Data Extraction

A standardised data extraction framework was developed prior to data extraction to ensure consistency across included studies. Data extraction was performed by a single reviewer using the predefined framework. Extracted variables included author and year of publication, country of origin, study design, study population, sample size, intervention or exposure, comparator where applicable, nutritional and body composition outcomes, wound healing and postoperative outcomes, perioperative optimisation strategies, and principal study findings. Extracted data were organised into a structured evidence table to facilitate comparison across studies and identify recurring patterns within the literature. The extracted data were also reviewed for completeness and consistency before synthesis.

### 2.6. Data Synthesis

Given the heterogeneity of study designs, populations, interventions, and outcome measures, quantitative meta-analysis was not performed. Instead, a narrative synthesis approach was undertaken.

Following data extraction, studies were grouped according to recurring concepts and patterns within the literature. Four major themes were identified:

1. Nutritional and body composition changes associated with GLP-1RAs.
2. Effects of malnutrition, sarcopenia, myosteatosis, and micronutrient deficiencies on surgical outcomes.
3. Postoperative outcomes associated with GLP-1RA use.
  - Clinical evidence of postoperative outcomes.
  - Mechanistic evidence relevant to wound healing and tissue repair.
4. Perioperative nutritional assessment and optimisation strategies.

Findings within each theme were synthesised descriptively to identify areas of consensus, conflicting evidence, and gaps in the current literature. Particular attention was given to the interaction between GLP-1RA therapy, nutritional status, and perioperative outcomes, with the aim of informing future nutritional optimisation strategies for surgical patients.

As this review aimed to map the breadth and characteristics of the available evidence rather than evaluate the certainty of effect estimates, a formal methodological quality or risk-of-bias assessment was not undertaken. This approach is consistent with the objectives and reporting recommendations of the Preferred Reporting Items for Systematic Reviews and Meta-Analyses Extension for Scoping Reviews (PRISMA-ScR), which does not require formal critical appraisal for scoping reviews.

## 3. Results

### 3.1. Nutritional and Body Composition Changes Associated with GLP-1RAs

Theme 1 summarises studies directly evaluating the effects of GLP-1RA therapy on nutritional status and body composition across five included studies comprising narrative reviews, observational studies and a systematic review with network meta-analysis. These studies provide direct evidence that GLP-1RA therapy is associated with nutritional and body composition changes but do not directly evaluate postoperative outcomes. Nutritional and body composition alterations are among the most consistently reported findings within the literature, with studies describing reduced energy intake, inadequate protein consumption, micronutrient deficiencies and loss of lean body mass following GLP-1RA initiation. These changes are primarily attributed to appetite suppression and reduced food intake, which contribute to substantial weight loss but may also increase the risk of nutritional inadequacy and sarcopenia. Given the established importance of adequate nutritional status for tissue repair, immune function and postoperative recovery, these findings identify potential perioperative nutritional risk factors that provide important context for interpreting subsequent evidence regarding surgical outcomes.

Reduced energy intake is consistently identified as a central mechanism underlying nutritional changes associated with GLP-1RA therapy. Urbina et al. describe appetite suppression, decreased food consumption and rapid weight loss as the principal drivers of nutritional vulnerability among patients receiving GLP-1RA. While these effects contribute to therapeutic weight reduction, they may also limit overall dietary intake and increase the risk of inadequate nutrient consumption. Similar concerns are reflected in direct dietary assessment studies. In a cross-sectional study of 69 GLP-1RA users, Johnson et al. report inadequate consumption across multiple food groups, including fruits, vegetables, grains and dairy products, suggesting that reductions in appetite may extend beyond caloric restriction to affect overall dietary quality. Despite these nutritional risks, only 20% of participants report referral to a dietitian and fewer than half receive guidance regarding management of gastrointestinal side effects that may further impair intake. Consistent with these findings, Butsch et al. demonstrate a progressive increase in nutritional deficiencies following GLP-1RA initiation, supporting the proposition that sustained reductions in dietary intake may contribute to clinically significant nutritional inadequacy over time. Together, these studies identify reduced energy intake as a commonly reported finding among patients receiving GLP-1RA therapy and a likely contributor to the nutritional and body composition changes reported throughout the literature.

Protein inadequacy emerges as a recurring concern among patients receiving GLP-1RA therapy and may represent an important consequence of sustained reductions in dietary intake. In a cross-sectional study of 69 GLP-1RA users, Johnson et al. report a mean protein intake of 77.3 ± 29.2 g/day. While this intake falls within the broad range of protein targets commonly recommended during weight loss, the substantial variability between participants suggests that many individuals may still fail to achieve adequate protein intake to optimise preservation of lean body mass. Similar concerns are highlighted by Mehta et al., who identify protein deficiency as a major contributor to impaired wound healing due to its role in collagen synthesis, angiogenesis and immune function [9]. The authors note that many patients undergoing significant weight loss fail to achieve recommended protein targets despite guidance recommending approximately 60–120 g of protein per day. Urbina et al. likewise identify inadequate protein intake as a key nutritional risk associated with GLP-1RA therapy, noting that appetite suppression and reduced food consumption may contribute to loss of lean body mass and increased sarcopenia risk when protein requirements are not met. These findings indicate that reductions in overall dietary intake may be accompanied by inadequate protein consumption, highlighting protein preservation as an important consideration during GLP-1RA-induced weight loss.

Micronutrient deficiencies are frequently reported among patients receiving GLP-1RA therapy and represent a recurring concern throughout the included literature. Urbina et al. identify deficiencies in vitamin D, vitamin B12, iron and folate as common nutritional consequences of appetite suppression, reduced dietary intake and rapid weight loss. Similar findings are reported by Butsch et al., who demonstrate that nutritional deficiencies become increasingly prevalent following GLP-1RA initiation, affecting approximately 12.7% of patients at six months and 22.4% at twelve months. Vitamin D deficiency is the most commonly identified deficiency, occurring in 7.5% of patients at six months and 13.6% at twelve months, while increased rates of B-vitamin and thiamine deficiencies are also observed [10]. Direct dietary assessment further supports these findings. Johnson et al. report inadequate intake of multiple micronutrients, including calcium, iron, magnesium, potassium and vitamins A, C, D and E among current GLP-1RA users. The identification of inadequate vitamin C intake is particularly relevant given its essential role in collagen synthesis and wound healing. In addition to vitamin C, other micronutrients involved in tissue repair, including zinc and copper, may have important perioperative implications when dietary intake is reduced. While deficiencies in vitamin D, vitamin B12, iron and folate are most frequently reported, the prevalence of deficiencies in nutrients with larger physiological stores may be underestimated in shorter-duration studies. Consequently, the long-term nutritional consequences of sustained GLP-1RA therapy may become increasingly apparent with prolonged treatment and warrant further investigation. Although the severity of deficiency varies between studies, the available evidence consistently demonstrates an increased risk of micronutrient inadequacy following GLP-1RA initiation, particularly when nutritional intake is not actively monitored or supplemented.

Loss of lean body mass and the potential development of sarcopenia are among the most clinically significant body composition changes associated with GLP-1RA therapy. The strongest evidence is provided by Karakasis et al., whose systematic review and network meta-analysis of 22 randomised controlled trials involving 2258 participants demonstrates that GLP-1RAs significantly reduce total body weight, fat mass and lean body mass. While reductions in adiposity account for the majority of weight loss, approximately 25% of total weight lost consists of lean mass, highlighting that weight reduction achieved with GLP-1RA therapy is not exclusively attributable to fat loss. Importantly, differences are observed between individual agents. Semaglutide 2.4 mg weekly and tirzepatide (which is a dual glucose-dependent insulinotropic polypeptide (GIP)/GLP-1RA) 15 mg weekly produce the greatest reductions in body weight and fat mass but are among the least effective at preserving lean tissue. In contrast, liraglutide is the only GLP-1RA associated with significant weight loss without a statistically significant reduction in lean mass. Similar concerns regarding muscle preservation are identified by Urbina et al., who highlight reductions in fat-free mass and increased sarcopenia risk as potential consequences of rapid weight loss, particularly when accompanied by inadequate protein intake. Butsch et al. likewise report that GLP-1-induced weight loss is not exclusively attributable to fat loss and may be accompanied by progressive skeletal muscle loss.

The nutritional and body composition changes identified in this theme have important implications within surgical populations. Reduced energy intake, inadequate protein consumption, micronutrient deficiencies and loss of lean body mass are all recognised risk factors for impaired tissue repair and recovery. Consequently, understanding the effects of these abnormalities on postoperative outcomes is critical when considering the perioperative implications of GLP-1RA therapy. The following theme therefore examines the relationship between malnutrition, sarcopenia, myosteatosis and micronutrient deficiencies and surgical outcomes.

### 3.2. Effects of Malnutrition, Sarcopenia and Micronutrient Deficiencies on Surgical Outcomes

Theme 2 summarises studies examining the relationship between malnutrition, sarcopenia, myosteatosis and micronutrient deficiencies and postoperative outcomes across a range of surgical populations. Importantly, these studies were not conducted specifically in patients receiving GLP-1RA therapy. Rather, they provide indirect evidence supporting biologically plausible mechanisms through which GLP-1RA-associated nutritional and body composition changes may influence perioperative recovery. Outcomes assessed included wound healing, infectious complications, major postoperative morbidity, length of hospital stay, mortality and long-term survival. Collectively, the available evidence consistently demonstrates that impaired nutritional status and adverse body composition characteristics are associated with poorer surgical outcomes, providing important context for interpreting the potential perioperative implications of GLP-1RA-associated nutritional changes.

Malnutrition is consistently associated with poorer postoperative outcomes across a range of surgical populations. In a prospective cohort study of 102 patients undergoing cytoreductive surgery and hyperthermic intraperitoneal chemotherapy (CRS/HIPEC), Reece et al. report that 34 patients (33%) are classified as malnourished prior to surgery. Malnourished patients are significantly more likely to experience postoperative infectious complications and demonstrate higher rates of preoperative weight loss and gastrointestinal symptoms than well-nourished patients. Increasing severity of malnutrition is also associated with prolonged hospitalisation, with length of stay increasing by an average of 7.65 days for each worsening category of nutritional status. Similar findings are reported by Seth et al., who identify malnutrition as an important contributor to impaired wound healing, wound dehiscence, skin breakdown, postoperative infection and increased postoperative mortality [11]. The review additionally highlights an association between malnutrition and prolonged hospital stay, which may further increase the risk of secondary complications such as venous thromboembolism, hospital-acquired pneumonia and urinary tract infections. These findings indicate that malnutrition adversely affects multiple aspects of postoperative recovery and is consistently associated with increased postoperative morbidity across diverse surgical settings.

Low skeletal muscle mass is consistently associated with poorer postoperative outcomes and reduced long-term survival across the included studies. The strongest pooled evidence is provided by Thormann et al., whose meta-analysis of 24 studies involving 5267 patients undergoing surgery for liver malignancies demonstrates that low skeletal muscle mass is associated with significantly higher odds of major postoperative complications (OR 1.56, 95% CI 1.25–1.95, *p* < 0.001). Patients with low skeletal muscle mass also experience significantly longer hospital stays, with a mean increase of 0.65 days compared with patients with preserved muscle mass. Although no statistically significant association is observed for overall postoperative complications or postoperative mortality, the findings indicate an increased risk of major postoperative morbidity among patients with reduced skeletal muscle reserves.

Similar findings are reported by Noguchi et al. in patients undergoing resection and free flap reconstruction for oral squamous cell carcinoma [12]. Using preoperative CT imaging, the authors demonstrate significantly poorer overall survival among patients with low skeletal muscle mass, with survival rates of 60.2% compared with 81.1% in patients with higher muscle mass. Low skeletal muscle mass remains an independent predictor of poorer survival on multivariable analysis (HR 2.339, 95% CI 1.008–5.429, *p* = 0.015). Wound-related complications are also common within this cohort, with surgical site infection occurring in 12.0% of patients and wound dehiscence or delayed wound healing occurring in 23.9%. Together, these findings demonstrate that low skeletal muscle mass is associated with increased postoperative morbidity and poorer survival outcomes across multiple surgical populations.

Myosteatosis is also associated with adverse postoperative outcomes. In a retrospective cohort study of 144 patients undergoing surgery for colorectal cancer, Giudici et al. evaluate the relationship between myosteatosis and postoperative morbidity. Patients with myosteatosis experience significantly higher rates of postoperative complications, with 65.2% developing postoperative morbidity compared with 39.1% of patients without myosteatosis (*p* = 0.002). Myosteatosis is additionally associated with prolonged hospitalisation, with affected patients remaining in hospital for a median of 11 days compared with 8 days among patients without myosteatosis (*p* = 0.001). In contrast, no significant association is identified between low skeletal muscle mass and postoperative complications within the same cohort. These findings identify myosteatosis as a significant predictor of postoperative morbidity and prolonged hospitalisation following colorectal cancer surgery [13].

Micronutrient deficiencies are also associated with adverse surgical outcomes. The strongest evidence is provided by Iglar et al., whose systematic review of 31 studies evaluates the relationship between vitamin D status and postoperative outcomes across a range of surgical specialties. Twenty-six of the 31 included studies (84%) report an association between low vitamin D levels and adverse surgical outcomes. Reported complications include increased rates of postoperative infection, impaired wound healing, longer hospital stays and poorer functional recovery. Similar findings are described by Seth et al., who identify vitamin and micronutrient deficiencies as important contributors to impaired tissue repair and postoperative morbidity. The authors highlight the role of micronutrients in collagen synthesis, immune function, angiogenesis and wound healing, noting that deficiencies may contribute to wound dehiscence, delayed healing and increased susceptibility to postoperative infection. These findings demonstrate a consistent association between micronutrient deficiencies and poorer postoperative outcomes across diverse surgical populations.

The evidence presented within this theme consistently demonstrates an association between nutritional status, body composition and postoperative outcomes. Malnutrition is associated with increased postoperative morbidity, infectious complications and prolonged hospitalisation, while low skeletal muscle mass is linked to higher rates of major postoperative complications and poorer long-term survival. Similarly, myosteatosis and micronutrient deficiencies are associated with adverse postoperative outcomes across multiple surgical populations. Importantly, many of these same abnormalities were identified in the preceding theme as potential consequences of GLP-1RA therapy. This overlap raises important questions regarding whether GLP-1-associated nutritional and body composition changes translate into measurable differences in postoperative outcomes. The following theme therefore examines clinical studies investigating postoperative outcomes among patients receiving perioperative GLP-1RA therapy.

### 3.3. GLP-1RA Use and Postoperative Outcomes

#### 3.3.1. Clinical Evidence of Postoperative Outcomes Associated with GLP-1RA Therapy

Theme 3A summarises studies directly evaluating postoperative outcomes among patients receiving GLP-1RA therapy across plastic surgery, hand surgery and arthroplasty populations. Included studies comprised retrospective cohort studies and one systematic review with meta-analysis. Outcomes assessed included wound-healing complications, wound dehiscence, surgical-site infection, periprosthetic joint infection, revision surgery and length of hospital stay. Collectively, these studies provide the strongest direct clinical evidence regarding the perioperative effects of GLP-1RA therapy. Overall, findings were heterogeneous, with adverse wound-healing signals reported in selected plastic surgery populations, whereas neutral or favourable postoperative outcomes were generally observed in hand surgery and arthroplasty cohorts.

Studies examining wound-healing complications reported differing findings across surgical settings. Adverse wound-healing outcomes were most consistently observed in plastic surgery cohorts undergoing body-contouring procedures. In a propensity-matched cohort study of patients undergoing panniculectomy, abdominoplasty and breast reduction, Lee et al. reported higher rates of wound-healing complications among patients receiving GLP-1RA across all three procedures [14]. The largest difference was observed following abdominoplasty, where wound-healing complications occurred in 9.8% of GLP-1RA users compared with 3.6% of matched controls. Similar findings were reported following panniculectomy (4.7% vs. 2.7%) and breast reduction surgery (2.6% vs. 1.3%). Patients with diabetes, peripheral vascular disease, smoking history, previous malignancy, and nutritional or endocrine disorders were excluded from the analysis, and cohorts were matched for BMI, reducing the likelihood that these factors accounted for the observed differences.

In contrast, comparable adverse findings were not observed in hand surgery populations. Bank et al. examined 303,360 patients undergoing carpal tunnel release, including 13,439 patients receiving perioperative GLP-1RA therapy. Although lower odds of wound dehiscence were reported among exposed patients, the clinical significance of this finding was uncertain given the modest effect size despite the large study population. Importantly, postoperative infection and scarring rates remained comparable between groups, and no increase in wound-related complications was observed among patients receiving GLP-1RA. These findings suggest that postoperative wound-healing outcomes may differ according to surgical setting, with adverse associations identified in body-contouring procedures but not following carpal tunnel release [15].

Postoperative infection outcomes were similarly reassuring. A systematic review and meta-analysis by Chan et al. synthesised eight retrospective cohort studies involving 76,091 patients undergoing arthroplasty. GLP-1RA use was associated with significantly lower rates of periprosthetic joint infection at both 90 days (OR 0.73) and two years (OR 0.72) following surgery. However, causality cannot be inferred from these findings. Given the established relationship between obesity and postoperative infection risk, it is possible that the observed association reflects improvements in body weight or metabolic health rather than a direct protective effect of GLP-1RA therapy. No significant differences were identified in wound dehiscence, periprosthetic fracture, aseptic loosening, revision surgery or length of hospital stay [16].

Beyond wound-healing and infectious complications, broader postoperative outcomes were largely comparable between exposed and unexposed patients. Verhey et al. reported no increase in postoperative medical complications among non-diabetic patients receiving GLP-1RA for weight loss before primary total hip arthroplasty. Likewise, no differences were identified in periprosthetic fracture, implant loosening, instability, dislocation or all-cause revision surgery. Similar findings emerged from the meta-analysis conducted by Chan et al., which likewise identified no significant differences in revision surgery, aseptic loosening, periprosthetic fracture or length of hospital stay between treatment groups. Consistent findings across both pooled and primary data suggest that GLP-1RA exposure is not associated with deterioration in major arthroplasty outcomes [16,17].

Findings from hand surgery further supported this overall trend. Bank et al. additionally reported lower odds of repeat carpal tunnel release within one year of surgery (OR 0.897, 95% CI 0.839–0.959) and substantially lower odds of median nerve injury (OR 0.399, 95% CI 0.192–0.832), while postoperative infection, scarring and wound complications remained comparable between treatment groups. The analysis adjusted for BMI, reducing the likelihood that obesity alone explained the observed findings.

Overall, the available clinical evidence suggests that postoperative outcomes associated with GLP-1RA therapy vary according to surgical population and outcome assessed. While studies conducted in arthroplasty and hand surgery populations generally report neutral or favourable postoperative outcomes, evidence from body-contouring procedures suggests a possible increase in wound-healing complications. These findings indicate that current direct clinical evidence does not support a uniform effect of GLP-1RA therapy across all surgical settings.

#### 3.3.2. Mechanistic Evidence Relevant to Wound Healing and Tissue Repair

In addition to direct clinical studies, several mechanistic and experimental investigations provide biological insights into how GLP-1RA therapy may influence wound healing and tissue repair. Importantly, these studies do not evaluate postoperative outcomes directly but instead propose biological mechanisms that may contribute to the clinical observations described above. Consequently, these findings should be interpreted as hypothesis-generating rather than evidence of clinical effect.

Experimental studies suggest that GLP-1RA therapy may enhance wound healing through anti-inflammatory and pro-angiogenic mechanisms. Proposed pathways include suppression of inflammatory signalling, enhanced angiogenesis, improved endothelial function and promotion of cellular repair processes. Experimental models have demonstrated accelerated wound closure, reduced inflammatory responses and enhanced neovascularisation following GLP-1RA treatment, providing biologically plausible mechanisms that may contribute to the favourable postoperative outcomes reported in some clinical studies. However, most supporting evidence is derived from experimental models rather than surgical patient populations, and the clinical significance of these mechanisms remains uncertain [18,19].

Conversely, Paschou et al. proposed several theoretical mechanisms through which GLP-1RA may adversely affect soft-tissue quality and skin integrity. The authors suggest that GLP-1 receptor activation within adipose-derived stem cells and fibroblasts may alter cellular metabolism, reduce ATP production, promote apoptosis and increase oxidative stress. Additional proposed mechanisms include reduced local oestrogen production within dermal white adipose tissue, potentially affecting fibroblast function, collagen synthesis and tissue maintenance. Collectively, these pathways have been proposed as possible contributors to soft-tissue volume loss, skin laxity and the phenomenon commonly described as “Ozempic face” [20].

Importantly, Paschou et al. also describe competing mechanisms that may exert protective effects on tissue integrity. GLP-1RA therapy has been proposed to reduce advanced glycation end-products, inhibit RAGE-mediated NF-κB activation, decrease inflammatory cytokine production and reduce oxidative stress, while limiting matrix metalloproteinase activity and supporting collagen homeostasis. Consequently, both potentially beneficial and detrimental biological pathways have been described, and the net effect of GLP-1RA therapy on tissue repair remains uncertain [20].

Taken together, the available mechanistic evidence provides biologically plausible explanations for both favourable and adverse postoperative findings observed across clinical studies. However, because these mechanisms are derived predominantly from experimental models and theoretical frameworks rather than prospective surgical cohorts, they should be interpreted cautiously until validated in well-designed clinical studies.

### 3.4. Perioperative Nutritional Assessment and Optimisation Strategies

Theme 4 summarises studies evaluating perioperative nutritional assessment and optimisation strategies relevant to surgical patients. Importantly, most of these interventions have not been evaluated specifically in patients receiving GLP-1RA therapy. Rather, they provide indirect evidence regarding nutritional approaches that may be applicable to this population. The included studies examined a range of strategies aimed at identifying nutritional risk and improving nutritional status before surgery, including formal nutritional screening, dietitian involvement, optimisation of protein and micronutrient intake, and multimodal prehabilitation programmes. Although the available evidence suggests these approaches may support wound healing, physiological reserve and postoperative recovery in selected surgical populations, direct evidence supporting their effectiveness in patients receiving GLP-1RA therapy remains limited.

Early identification of nutritional risk is frequently highlighted as an important component of perioperative care. In a prospective cohort study of 102 patients undergoing cytoreductive surgery and hyperthermic intraperitoneal chemotherapy, Reece et al. utilised the Subjective Global Assessment (SGA) to evaluate preoperative nutritional status and identified malnutrition in 34 patients (33%) prior to surgery. Patients classified as malnourished experienced higher rates of postoperative infectious complications and significantly longer hospital stays, demonstrating the association between poor nutritional status and adverse postoperative outcomes. Similar recommendations are reported by Penny et al., who identify nutritional screening as an important step in wound management and emphasise the value of detecting protein and micronutrient deficiencies before the development of wound-healing complications [21]. Collectively, these findings suggest that structured nutritional assessment tools may assist in identifying patients at nutritional risk and could facilitate earlier nutritional intervention within the perioperative setting.

Despite the nutritional risks associated with GLP-1RA therapy, dietetic involvement appears limited. In a cross-sectional study of 69 GLP-1RA users, Johnson et al. reported that only 20% of participants had been referred to a dietitian despite widespread evidence of inadequate protein intake and multiple micronutrient inadequacies. Furthermore, only 51% of participants reported receiving guidance regarding management of treatment-related gastrointestinal side effects that may further impair dietary intake. These findings suggest that opportunities for nutritional intervention may be underutilised among patients receiving GLP-1RA therapy and indicate a potential role for dietitians in identifying and addressing nutritional inadequacies before surgery [22].

Protein optimisation is frequently proposed as a potentially important component of perioperative nutritional management. In a narrative review examining patients undergoing body-contouring surgery following massive weight loss, Mehta et al. identified protein deficiency as a major contributor to impaired wound healing because of its role in collagen synthesis, angiogenesis and immune function. The authors noted that many post-weight-loss patients failed to achieve recommended protein targets despite guidance recommending approximately 60–120 g of protein per day. To address this risk, the review proposed adequate caloric intake, optimisation of protein consumption, distribution of protein intake throughout the day and the use of small, frequent meals to maximise nutritional intake. Similar findings were reported by Penny et al., who described protein as essential throughout all stages of wound healing, supporting fibroblast proliferation, collagen deposition, angiogenesis, tissue remodelling and immune function. The authors suggested protein intakes of 1–2 g/kg/day for patients with wounds and noted that individuals with hard-to-heal wounds may require substantially higher protein requirements. Collectively, these findings suggest that maintaining adequate protein intake may represent an important consideration for supporting wound healing and preserving lean body mass during the perioperative period.

Preservation of lean body mass may represent an additional consideration for perioperative nutritional optimisation. As discussed in Theme 2, reduced skeletal muscle mass has consistently been associated with increased postoperative morbidity, prolonged hospitalisation and poorer long-term survival across multiple surgical populations. Given that GLP-1RA therapy has been associated with reductions in lean body mass, strategies aimed at preserving muscle quantity and quality may be particularly relevant during periods of weight loss. Adequate protein intake, resistance exercise and multimodal prehabilitation programmes have all been proposed as approaches to minimise muscle loss and maintain functional reserve prior to surgery. However, prospective studies are required to determine whether interventions targeting skeletal muscle preservation improve postoperative outcomes in patients receiving GLP-1RA therapy.

Correction of micronutrient deficiencies has also been proposed as a component of perioperative nutritional optimisation. Penny et al. describe several micronutrients as important contributors to wound healing and tissue repair. Vitamin A supports immune function, re-epithelialisation and collagen stability, while vitamin C is required for collagen synthesis, fibroblast proliferation and wound matrix formation. Vitamin D is reported to exert immunomodulatory effects through regulation of inflammatory pathways and may contribute to tissue integrity and infection prevention. Trace elements are similarly identified as important components of wound healing. Zinc supports extracellular matrix remodelling, antioxidant defence and tissue turnover, whereas copper contributes to collagen cross-linking, angiogenesis and vascular endothelial growth factor signalling. Given the established associations between micronutrient deficiencies and adverse surgical outcomes, identification and correction of deficiencies have been proposed as potential components of perioperative nutritional optimisation. However, direct evidence supporting these strategies in patients receiving GLP-1RA therapy remains limited.

Multimodal prehabilitation programmes combining nutritional support and exercise have also emerged as a potential strategy to improve preoperative physiological reserve. In a systematic review of patients undergoing lung cancer surgery, Ferreira et al. evaluated both nutrition-only interventions and multimodal prehabilitation programmes incorporating nutrition and exercise. Across the included studies, multimodal prehabilitation was associated with improvements in functional capacity and physical fitness before surgery. One randomised trial demonstrated a 37.7 m greater improvement in six-minute walk distance compared with controls, while a secondary analysis reported improvements in physical fitness among 60% of patients undergoing prehabilitation compared with 21% of control patients (*p* < 0.001). Evidence demonstrating reductions in postoperative complications was more limited, with only the nutrition-only intervention study reporting a significant reduction in postoperative morbidity. Consequently, while multimodal prehabilitation may improve functional capacity and physiological reserve before surgery, its impact on postoperative outcomes, particularly among patients receiving GLP-1RA therapy, remains uncertain [23].

The studies included within this theme identify several nutritional assessment and optimisation strategies that have been proposed to improve perioperative care across a range of surgical populations. These include formal nutritional screening, dietitian involvement, optimisation of protein intake, correction of micronutrient deficiencies and multimodal prehabilitation. While these approaches may help address nutritional and body composition abnormalities associated with poorer surgical outcomes, most have not been evaluated specifically in patients receiving GLP-1RA therapy. Consequently, these strategies should be considered as proposed perioperative considerations rather than evidence-based recommendations for this population. Future prospective interventional studies are required to determine whether targeted nutritional optimisation improves postoperative outcomes in patients receiving GLP-1RA therapy.

## 4. Discussion

This scoping review identified a complex relationship between GLP-1RA therapy, nutritional status and postoperative outcomes. The most consistent finding across the available literature was that GLP-1RA therapy is associated with nutritional and body composition changes, including reduced energy intake, inadequate protein consumption, micronutrient deficiencies and loss of lean body mass. These findings are particularly relevant within surgical populations because many of the same nutritional abnormalities have independently been associated with poorer postoperative outcomes. However, despite this recognised risk profile, studies directly evaluating postoperative outcomes among patients receiving GLP-1RA generally reported neutral or favourable findings, although important differences were observed between surgical specialties. This apparent disconnect represents the central finding of the present review and highlights the complexity of evaluating the perioperative implications of GLP-1RA therapy. Rather than reflecting a single beneficial or detrimental effect, the available evidence suggests that postoperative outcomes are likely influenced by the interaction between the metabolic benefits of GLP-1RA, treatment-related nutritional changes and procedure-specific surgical factors.

One of the most striking findings of this review was the remarkable consistency with which GLP-1RA therapy was associated with nutritional and body composition changes. In contrast to the heterogeneous evidence surrounding postoperative outcomes, studies evaluating nutritional status consistently reported reduced energy intake, inadequate protein consumption, micronutrient deficiencies and loss of lean body mass. These findings are biologically plausible given that the therapeutic efficacy of GLP-1RA is largely mediated through appetite suppression and reduced caloric intake. While these mechanisms facilitate clinically meaningful weight loss and improvements in metabolic health, they may also reduce overall dietary intake and increase the likelihood of nutritional inadequacy if nutritional intake is not actively monitored.

Particularly noteworthy was the recurring evidence of inadequate protein intake and progressive reductions in lean body mass. Approximately one quarter of total weight loss associated with GLP-1RA therapy has been reported to occur through loss of lean tissue rather than adipose tissue alone, raising important questions regarding preservation of skeletal muscle during treatment. This finding is clinically relevant because skeletal muscle serves not only as a determinant of physical function but also as an important metabolic reserve during periods of physiological stress. Likewise, deficiencies in micronutrients essential for immune function, collagen synthesis and tissue repair—including vitamin D, vitamin B12, iron and folate—were repeatedly identified across multiple studies. Collectively, these findings suggest that the nutritional and body composition changes associated with GLP-1RA therapy extend beyond reductions in adiposity and may result in nutritional vulnerabilities that warrant consideration in patients undergoing surgery. Importantly, the consistency of these findings across multiple study designs and patient populations suggests that nutritional compromise may represent a recurring finding of GLP-1RA therapy.

The nutritional and body composition changes identified in this review are particularly relevant because they closely mirror many of the established predictors of adverse postoperative outcomes. Across diverse surgical populations, malnutrition, sarcopenia, myosteatosis and micronutrient deficiencies have consistently been associated with increased postoperative complications, prolonged hospitalisation and impaired recovery. Malnutrition has repeatedly been associated with higher rates of postoperative infection, while low skeletal muscle mass has been linked to increased major postoperative complications, longer hospital stays and poorer long-term survival. Emerging evidence also suggests that myosteatosis may be a stronger predictor of postoperative morbidity than skeletal muscle quantity alone, emphasising the importance of muscle quality in addition to muscle mass. Similarly, deficiencies in micronutrients involved in immune function, collagen synthesis and tissue repair have consistently been associated with impaired wound healing and increased postoperative morbidity across multiple surgical specialties. These findings reinforce the well-established importance of nutritional status as a determinant of surgical recovery.

When considered together, the evidence presented in Themes 1 and 2 identifies an apparent paradox. GLP-1RA therapy has been consistently associated with nutritional and body composition changes that have traditionally been associated with poorer postoperative outcomes, yet the available clinical studies generally do not demonstrate a corresponding increase in postoperative morbidity. Several explanations may account for this apparent discrepancy. The metabolic benefits of GLP-1RA therapy, including improvements in obesity-related comorbidity, glycaemic control and systemic inflammation, may offset some of the potential adverse effects associated with nutritional compromise. Conversely, the nutritional changes observed may not reach a threshold sufficient to adversely influence postoperative recovery in many patients, particularly when treatment duration is relatively short or nutritional intake is maintained. It is also possible that current observational studies are insufficiently sensitive to detect clinically meaningful nutritional effects because of heterogeneity in patient populations, surgical procedures and study design. Consequently, the relationship between GLP-1RA-associated nutritional changes and postoperative outcomes is unlikely to be linear and probably reflects the balance between competing beneficial and detrimental physiological effects rather than a single dominant mechanism.

The available clinical evidence examining postoperative outcomes associated with GLP-1RA therapy was generally reassuring but should be interpreted cautiously. Across multiple surgical populations, GLP-1RA therapy was not consistently associated with increased postoperative complications, with several studies reporting either neutral outcomes or reductions in complications such as wound dehiscence and periprosthetic joint infection. However, these findings were not universal. Evidence from plastic surgery populations suggested a potential increase in wound-healing complications following body-contouring procedures, whereas studies involving hand surgery and arthroplasty generally reported neutral or favourable outcomes. Collectively, these findings suggest that the perioperative effects of GLP-1RA therapy are unlikely to be uniform across all surgical settings and instead may depend on the interaction between patient characteristics, nutritional status and the physiological demands of the surgical procedure.

Interpretation of the available evidence should also consider several important clinical modifiers that remain incompletely characterised within the current literature. These include the specific GLP-1RA administered, dose, treatment duration, indication for therapy, baseline BMI, diabetes status, magnitude and rate of weight loss, baseline nutritional status, gastrointestinal adverse effects, and adherence to nutritional support strategies such as adequate protein intake and resistance exercise. Each of these factors has the potential to influence perioperative nutritional status, body composition and postoperative recovery, yet few available studies have evaluated their independent contributions. The duration and intensity of GLP-1RA therapy may be particularly important, as prolonged treatment and higher cumulative exposure could increase the likelihood of nutritional deficiencies and progressive lean body mass loss. Similarly, patients receiving GLP-1RA for obesity may differ substantially from those treated for type 2 diabetes with respect to baseline metabolic health, nutritional status and perioperative risk. However, few available studies reported treatment duration, cumulative dose or treatment indication in sufficient detail to determine whether these factors independently modify postoperative outcomes.

Furthermore, individual GLP-1RAs should not necessarily be considered interchangeable. Evidence from the present review suggests that semaglutide and tirzepatide (which is a dual glucose-dependent insulinotropic polypeptide (GIP)/GLP-1RA) are associated with greater reductions in total body weight and lean body mass than liraglutide, indicating that the nutritional consequences of therapy may differ between agents. Whether these pharmacological differences translate into clinically meaningful differences in perioperative outcomes remains unknown because comparative surgical studies between individual GLP-1RAs are currently lacking. These differences may have important perioperative implications, particularly for patients undergoing prolonged treatment or experiencing rapid weight loss. Future studies should therefore evaluate individual GLP-1RAs separately rather than considering the drug class as a homogeneous exposure.

The heterogeneity of surgical procedures represented within the available literature further complicates interpretation of the current evidence. Procedures such as abdominoplasty, panniculectomy and breast reduction involve extensive soft-tissue dissection, disruption of local vascular supply, prolonged wound closure under tension and a substantial reliance on collagen synthesis, angiogenesis and tissue remodelling during recovery. Consequently, nutritional deficiencies and reductions in lean body mass may have greater clinical relevance in these procedures than in operations involving relatively limited soft-tissue disruption, such as carpal tunnel release. Similarly, arthroplasty populations differ substantially from reconstructive plastic surgery cohorts with respect to patient characteristics, mechanisms of postoperative complications and determinants of surgical success. Whereas successful wound healing may be the principal determinant of outcome following body-contouring procedures, arthroplasty outcomes may be influenced more strongly by obesity, metabolic control, implant-related factors and infection risk. These observations suggest that perioperative GLP-1RA therapy should not be considered a uniform exposure across all surgical specialties. Rather, procedure-specific risk stratification may be required, particularly for operations in which successful outcomes depend heavily on soft-tissue healing, collagen synthesis and adequate nutritional reserve.

Interpretation of the current evidence is further limited by the predominantly observational design of the available clinical studies. Patients receiving GLP-1RA therapy may differ systematically from untreated patients with respect to obesity severity, diabetes control, healthcare engagement, access to multidisciplinary care, preoperative optimisation and the magnitude of weight loss achieved before surgery. Consequently, the favourable postoperative outcomes reported in several arthroplasty and hand surgery studies may reflect improvements in metabolic health, reductions in obesity-related risk or residual confounding rather than direct effects of GLP-1RA therapy itself. Conversely, the adverse wound-healing outcomes reported in body-contouring surgery may similarly be influenced by recent weight loss, unmeasured nutritional deficiencies, altered body composition or other patient characteristics that were not fully accounted for despite propensity matching. Although several studies attempted to minimise confounding through matching or statistical adjustment, residual confounding remains difficult to exclude. These limitations highlight the need for prospective studies capable of more comprehensively characterising nutritional status, body composition and perioperative risk while accounting for these important patient- and treatment-related variables.

The heterogeneous postoperative outcomes observed across clinical studies may also reflect the complex and, at times, opposing biological effects of GLP-1RA therapy on wound healing and tissue repair. Experimental evidence suggests that GLP-1RA may exert several potentially beneficial effects on the wound-healing process through modulation of inflammatory pathways, enhancement of angiogenesis, improvement in endothelial function and promotion of cellular repair mechanisms. These effects provide biologically plausible explanations for the favourable postoperative outcomes reported in several arthroplasty and hand surgery studies. Conversely, mechanistic studies have also proposed pathways through which GLP-1RA may adversely influence soft-tissue quality, including alterations in fibroblast function, increased oxidative stress and disruption of collagen homeostasis. These competing biological mechanisms provide a plausible explanation for the apparent inconsistency of the current clinical evidence and reinforce the likelihood that postoperative outcomes are determined by the balance between the metabolic benefits of treatment, nutritional status and the biological requirements of individual surgical procedures rather than by a single direct pharmacological effect.

The findings of this review have several important implications for perioperative practice. Rather than suggesting that GLP-1RA therapy itself should routinely alter perioperative management, the available evidence highlights the importance of recognising and addressing the nutritional consequences that may accompany treatment. While current clinical studies generally do not demonstrate consistently worse postoperative outcomes among patients receiving GLP-1RA, nutritional and body composition changes—including reduced energy intake, inadequate protein intake, micronutrient deficiencies and loss of lean body mass—were consistently identified across the literature and have independently been associated with poorer surgical outcomes. Consequently, nutritional assessment may represent an important consideration during preoperative evaluation, particularly for patients undergoing prolonged treatment, experiencing substantial weight loss or undergoing procedures that rely heavily on soft-tissue healing.

Current perioperative assessment frequently focuses on comorbidities, functional status and procedural risk, whereas nutritional status and body composition may receive comparatively less attention. The findings of this review suggest that nutritional screening, assessment of dietary intake and consideration of body composition may provide additional information when evaluating patients receiving GLP-1RA therapy before surgery. Similarly, dietetic involvement, optimisation of protein intake, correction of identified micronutrient deficiencies and multimodal prehabilitation have all been proposed as strategies that may improve nutritional status and physiological reserve before surgery. However, it is important to emphasise that these approaches have not been evaluated specifically in patients receiving GLP-1RA therapy and therefore should be considered proposed perioperative considerations rather than evidence-based recommendations for this population. Whether targeted nutritional optimisation improves postoperative outcomes among patients receiving GLP-1RA remains uncertain and requires prospective evaluation.

Several important research priorities emerge from the current evidence. First, adequately powered prospective cohort studies are required to define the longitudinal relationship between GLP-1RA therapy, nutritional status, body composition and postoperative outcomes while accounting for important clinical modifiers such as diabetes status, baseline BMI, rate and magnitude of weight loss, nutritional status, gastrointestinal adverse effects and individual GLP-1RA. Second, randomised controlled trials evaluating perioperative nutritional optimisation strategies—including protein supplementation, micronutrient replacement, resistance exercise and multimodal prehabilitation—are required to determine whether targeted interventions improve postoperative outcomes in patients receiving GLP-1RA therapy. Third, future studies should adopt procedure-specific approaches to determine whether perioperative risk differs across surgical specialties, particularly between operations requiring extensive soft-tissue reconstruction and procedures such as arthroplasty or minimally invasive surgery. Finally, future research should focus on developing evidence-based perioperative pathways integrating nutritional assessment, body composition evaluation and targeted nutritional optimisation strategies tailored to patients receiving GLP-1RA therapy.

This review has several strengths. To our knowledge, it is the first scoping review to synthesise evidence examining the relationship between GLP-1RA therapy, nutritional status and perioperative surgical outcomes. By integrating evidence relating to GLP-1RA-associated nutritional and body composition changes, established nutritional risk factors for poor postoperative outcomes, direct clinical evidence evaluating perioperative GLP-1RA use and perioperative nutritional optimisation strategies, this review provides a broader conceptual framework through which the potential perioperative implications of GLP-1RA can be interpreted. In doing so, it highlights important evidence gaps and identifies priorities for future clinical research.

Several limitations should be considered when interpreting the findings of this review. First, the literature search was limited to a single database (PubMed), representing an important limitation of this review. Although PubMed provides extensive coverage of the biomedical literature and was considered appropriate for the exploratory objectives of this scoping review, restricting the search to a single database may have resulted in the omission of relevant studies indexed exclusively in other databases, such as Embase, Scopus or Web of Science. Consequently, it is possible that not all eligible evidence was captured, which may have influenced the comprehensiveness of the evidence synthesis and the identification of all relevant publications. Future reviews incorporating multiple databases would likely provide a more comprehensive representation of the available literature and strengthen the completeness of the evidence base. Second, study selection was conducted by a single reviewer rather than two independent reviewers. Although this approach is considered acceptable for scoping reviews and is consistent with the exploratory objective of mapping the available evidence, it may have increased the risk of missed eligible studies or subjective decisions during the screening process. Third, the included literature was highly heterogeneous with respect to study design, patient populations, surgical procedures and outcome measures, limiting direct comparison between studies. Fourth, most available evidence consisted of retrospective observational studies, limiting causal inference and increasing susceptibility to residual confounding and selection bias. Finally, although this review synthesised evidence relating to nutritional status and perioperative outcomes, formal methodological quality and risk-of-bias assessment was not undertaken, consistent with the objectives of a scoping review. Consequently, the findings should be interpreted as a mapping of the available evidence rather than an assessment of the certainty or strength of that evidence. Despite these limitations, this review provides a broad synthesis of the current literature and identifies several important directions for future investigation.

## 5. Conclusions

This scoping review suggests that the relationship between GLP-1RA therapy, nutritional status and surgical outcomes is multifaceted and remains incompletely understood. While current clinical evidence generally indicates that perioperative GLP-1RA use is not consistently associated with worse postoperative outcomes, these agents are repeatedly associated with nutritional and body composition changes, including reduced energy intake, protein inadequacy, micronutrient deficiencies and loss of lean body mass. Importantly, many of these same abnormalities have independently been associated with increased postoperative morbidity, impaired wound healing and prolonged recovery across diverse surgical populations.

The findings of this review suggest that nutritional changes associated with GLP-1RA therapy may represent an important consideration in perioperative management, although direct evidence linking these changes to postoperative outcomes remains limited. These findings support consideration of nutritional screening, body composition assessment and individualised nutritional optimisation strategies where clinically appropriate; however, prospective studies are required to determine whether these approaches improve postoperative outcomes in patients receiving GLP-1RA therapy.

Overall, the available evidence suggests that a more comprehensive perioperative approach incorporating both the metabolic benefits and potential nutritional consequences of GLP-1RA therapy may be beneficial. Future prospective studies are required to clarify the long-term relationship between GLP-1RA-induced nutritional changes and surgical outcomes, determine whether targeted nutritional interventions improve perioperative recovery, and inform the development of evidence-based perioperative management strategies for this growing patient population.

## Data Availability

No new data were created or analyzed in this study. Data sharing is not applicable to this article.

## Supplementary Materials

The following supporting information can be downloaded at: https://www.mdpi.com/article/10.3390/medicina62081434/s1.
