# Supplementary material for: Perioperative Nutrition and Surgical Outcomes in Patients Receiving Glucagon-like Peptide-1 Receptor Agonists: A Scoping Review
Source: Medicina (Kaunas). 2026 Jul 23;62(8):1434. doi: 10.3390/medicina62081434 (PMC13514910; doi:10.3390/medicina62081434)
Supplement: Supplementary file 1 [file medicina-62-01434-s001.zip › medicina-4422032-supplementary.pdf]

Supplementary Table S1

Theme 1. Nutritional and body composition changes associated with GLP-1 receptor agonists

Study	Study category	Design	Population (n)	GLP-1 agent	Indication	Dose / treatment duration	Comparator	Surgical specialty	Outcomes	Key findings	Directness of evidence	Major limitations
**Urbina et al. (2026)**	Clinical review	Narrative review	Patients receiving GLP-1 receptor agonists	Semaglutide, liraglutide, tirzepatide	Obesity and/or type 2 diabetes	Variable across included studies	Not applicable	Not applicable	Nutritional status, lean mass	Reduced dietary intake, protein inadequacy, micronutrient deficiencies and lean mass loss associated with GLP-1RA therapy.	Direct nutritional	Narrative review; no perioperative or surgical outcome data.
**Butsch et al. (2025)**	Clinical study	Retrospective observational study	Adults with type 2 diabetes receiving GLP-1 receptor agonists	Multiple GLP-1 receptor agonists	Type 2 diabetes	Up to 12 months following GLP-1RA initiation	Baseline nutritional status before GLP-1RA initiation	Not applicable	Nutritional deficiencies, muscle loss	Nutritional deficiencies increased following GLP-1RA initiation; vitamin D deficiency was most common, with	Direct nutritional	Observational design; limited nutritional assessment; no surgical outcomes.

Study	Study category	Design	Population (n)	GLP-1 agent	Indication	Dose / treatment duration	Comparator	Surgical specialty	Outcomes	Key findings	Directness of evidence	Major limitations
										increasing deficiencies over 12 months.		
**Mehta et al. (2025)**	Clinical review	Narrative review	Post-massive weight loss/body contouring patients	GLP-1 receptor agonists	Weight loss	Not reported	Not applicable	Plastic and reconstructive surgery (body contouring)	Protein deficiency, nutritional optimisation	Highlights protein deficiency and recommends perioperative nutritional optimisation strategies in patients undergoing body contouring after GLP-1RA-associated weight loss.	Indirect nutritional	Narrative review; recommendations largely based on expert opinion and indirect evidence.
**Karakasis et al. (2025)**	Clinical review	Systematic review and network	22 RCTs (2,258 participants)	Semaglutide, liraglutide, tirzepatide	Obesity, overweight and/or	Variable across included trials	Placebo or active comparator (varied	Not applicable	Body composition	Approximately 25% of total weight loss	Direct nutritional	No surgical outcomes; heterogeneity

Study	Study category	Design	Population (n)	GLP-1 agent	Indication	Dose / treatment duration	Comparator	Surgical specialty	Outcomes	Key findings	Directness of evidence	Major limitations
		meta-analysis		and other GLP-1 receptor agonists/co-agonists	type 2 diabetes		across included studies)			comprised lean mass; semaglutide and tirzepatide produced the greatest reductions in body weight and fat mass.		between included trials.
**Johnson et al. (2025)**	Clinical study	Cross-sectional study	69 GLP-1RA users	Multiple GLP-1 receptor agonists	Not reported	Not reported	Not applicable	Not applicable	Dietary intake	Participants demonstrated inadequate protein and micronutrient intake, with low rates of dietitian referral and nutritional counselling.	Direct nutritional	Small sample size; self-reported dietary intake; cross-sectional design.

Study	Design	Population (n)	Outcomes	Key findings	Evidence type	Major limitations

Theme 2. Nutritional abnormalities associated with surgical outcomes

Study	Study category	Design	Population (n)	GLP-1 agent	Indication	Dose / treatment duration	Comparator	Surgical specialty	Outcomes	Key findings	Directness of evidence	Major limitations
**Seth et al. (2024)**	Clinical review	Narrative review	Multiple surgical specialties	Not applicable	Not applicable	Not applicable	Not applicable	Multiple surgical specialties	Wound healing, postoperative complications	Malnutrition was consistently associated with impaired wound healing, increased postoperative complications, infections, prolonged hospital stay and poorer aesthetic outcomes.	Indirect clinical	Narrative review; conclusions based on heterogeneous literature and no quantitative synthesis.
**Reece et al. (2019)**	Clinical study	Prospective cohort	102 patients undergoing	Not applicable	Not applicable	Not applicable	Well-nourished versus malnourished patients	Surgical oncology (CRS/HIPEC)	Infectious complications, length of stay	Preoperative malnutrition was	Indirect clinical	Single-centre study; relatively

Study	Study category	Design	Population (n)	GLP-1 agent	Indication	Dose / treatment duration	Comparator	Surgical specialty	Outcomes	Key findings	Directness of evidence	Major limitations
			CRS/HIPEC							associated with significantly higher postoperative infectious complications and longer hospital stay.		small sample size.
**Noguchi et al. (2025)**	Clinical study	Retrospective cohort	92 patients undergoing free flap reconstruction for oral SCC	Not applicable	Not applicable	Not applicable	Low versus high skeletal muscle index	Oral and maxillofacial surgery (free flap reconstruction)	Surgical site infection, wound healing, overall survival	Low skeletal muscle mass was associated with poorer overall survival and adverse postoperative outcomes.	Indirect clinical	Single-centre retrospective study with limited sample size.

Study	Study category	Design	Population (n)	GLP-1 agent	Indication	Dose / treatment duration	Comparator	Surgical specialty	Outcomes	Key findings	Directness of evidence	Major limitations
**Iglar & Hogan (2015)**	Clinical review	Systematic review	31 studies	Not applicable	Not applicable	Not applicable	Variable across included studies	Multiple surgical specialties	Surgical outcomes	Twenty-six of 31 studies reported adverse postoperative outcomes associated with vitamin D deficiency.	Indirect clinical	Considerable heterogeneity across surgical specialties and study designs.
**Thormann et al. (2022)**	Clinical review	Meta-analysis	24 studies (5,267 patients)	Not applicable	Not applicable	Not applicable	Patients with low versus normal skeletal muscle mass	Hepatobiliary surgery	Major postoperative complications, length of stay, mortality	Low skeletal muscle mass significantly increased major postoperative complications and length of	Indirect clinical	Predominantly observational studies with variable definitions of sarcopenia.

Study	Study category	Design	Population (n)	GLP-1 agent	Indication	Dose / treatment duration	Comparator	Surgical specialty	Outcomes	Key findings	Directness of evidence	Major limitations
										hospital stay.		
**Giudici et al. (2025)**	Clinical study	Retrospective cohort	242 emergency laparotomy patients	Not applicable	Not applicable	Not applicable	Patients with versus without sarcopenia/myosteatosis	Emergency general surgery	ICU admission, complications, length of stay, mortality	Myosteatosis, rather than sarcopenia, independently predicted worse postoperative outcomes and prolonged hospital stay.	Indirect clinical	Single-centre retrospective study; emergency surgery population limits generalisability.

Theme 3. Postoperative outcomes associated with GLP-1 receptor agonists

Study	Study category	Design	Population (n)	GLP-1 agent	Indication	Dose / treatment duration	Comparator	Surgical specialty	Outcomes	Key findings	Directness of evidence	Major limitations
**Lee et al. (2025)**	Clinical study	Propensity score-matched retrospective cohort	Patients undergoing panniculectomy, abdominoplasty or breast reduction	GLP-1 receptor agonists (specific agents not reported)	Not reported	Preoperative GLP-1RA use; duration not reported	Non-GLP-1RA users	Plastic and reconstructive surgery	Surgical site wound complications	GLP-1RA use was associated with significantly higher wound complication rates following panniculectomy, abdominoplasty and breast reduction.	Direct clinical	Retrospective database study; specific GLP-1 agents and treatment duration not reported; residual confounding cannot be excluded despite propensity matching.
**Bank et al. (2025)**	Clinical study	Retrospective cohort	303,360 patients undergoing carpal tunnel release (13,439	GLP-1 receptor agonists (specific ic	Not reported	Perioperative GLP-1RA use; duration not reported	Non-GLP-1RA users	Hand surgery	Wound dehiscence, infection, scarring, revision surgery,	GLP-1RA use was associated with lower odds of wound dehiscence,	Direct clinical	Retrospective observational study; medication type and duration

Study	Study category	Design	Population (n)	GLP-1 agent	Indication	Dose / treatment duration	Comparator	Surgical specialty	Outcomes	Key findings	Directness of evidence	Major limitations
			GLP-1RA users)	agents not reported)					nerve injury	repeat surgery and median nerve injury, with no increase in infection or scarring.		not reported; administrative database limitations.
**Chan et al. (2025)**	Clinical review	Systematic review and meta-analysis	8 retrospective cohort studies (76,091 patients)	Multiple GLP-1 receptor agonists	Obesity and/or type 2 diabetes	Variable across included studies	Non-GLP-1RA users	Orthopaedic surgery (arthroplasty)	Periprosthetic joint infection, revision, wound complications, length of stay	GLP-1RA therapy reduced the risk of periprosthetic joint infection without increasing other postoperative complications.	Direct clinical	Meta-analysis of retrospective studies with heterogeneity in study populations, medications and perioperative management.
**Verhey et al. (2025)**	Clinical study	Retrospective cohort	Patients undergoing primary	GLP-1 receptor	Weight loss in non-	Not reported	Non-GLP-	Orthopaedic surgery (total hip	Medical complications,	GLP-1RA use was not associated	Direct clinical	Retrospective study; adjusted

Study	Study category	Design	Population (n)	GLP-1 agent	Indication	Dose / treatment duration	Comparator	Surgical specialty	Outcomes	Key findings	Directness of evidence	Major limitations
			total hip arthroplasty	agonists (specific agents not reported)	diabetic patients		1RA users	arthroplasty)	prosthetic joint infection, revision surgery, dislocation, fracture	with increased postoperative medical or surgical complications following total hip arthroplasty.		for obesity diagnosis rather than actual BMI; medication duration not reported.
**Ghebrehiwet-Kuflom et al. (2025)**	Preclinical mechanistic review	Narrative review	Experimental and preclinical studies	Multiple GLP-1 receptor agonists	Wound healing and tissue repair	Not applicable	Not applicable	Not applicable	Inflammation, angiogenesis, keratinocyte migration, endothelial function	GLP-1 receptor agonists may promote wound healing through anti-inflammatory and pro-angiogenic mechanisms, although evidence is predominantly preclinical.	Mechanistic	Narrative review based largely on experimental and animal studies without direct clinical outcome data.

Study	Study category	Design	Population (n)	GLP-1 agent	Indication	Dose / treatment duration	Comparator	Surgical specialty	Outcomes	Key findings	Directness of evidence	Major limitations
**Paschou et al. (2025)**	Preclinical mechanistic review	Narrative review	Mechanistic and translational studies	Multiple GLP-1 receptor agonists	Skin ageing and soft tissue biology	Not applicable	Not applicable	Not applicable	Dermal biology, collagen homeostasis, oxidative stress	GLP-1 receptor agonists may exert both protective and detrimental effects on skin biology through competing molecular pathways affecting fibroblasts, collagen synthesis and oxidative stress.	Mechanistic	Hypothesis-generating review with limited clinical evidence.
**Roan et al. (2017)**	Preclinical experimental study	Experimental animal study	Diabetic murine wound model	Exendin-4	Diabetes mellitus	Reported in original experimental protocol	Vehicle-treated controls	Not applicable	Wound closure, angiogenesis, inflammation	Exendin-4 accelerated wound healing, enhanced angiogenesi	Mechanistic	Animal model; findings may not directly translate

Study	Study category	Design	Population (n)	GLP-1 agent	Indication	Dose / treatment duration	Comparator	Surgical specialty	Outcomes	Key findings	Directness of evidence	Major limitations
										s and reduced inflammatory responses in diabetic wounds.		to human surgical patients.

Theme 4. Perioperative nutritional assessment and optimization

Study	Study category	Design	Population (n)	GLP-1 agent	Indication	Dose / treatment duration	Comparator	Surgical specialty	Outcomes	Key findings	Directness of evidence	Major limitations
**Ferreira et al. (2021)**	Clinical review	Systematic review	Five studies of patients undergoing lung cancer surgery	Not applicable	Not applicable	Not applicable	Standard care or usual perioperative management (varied across included studies)	Thoracic surgery	Functional capacity, postoperative complications, pulmonary function	Preoperative nutritional optimisation and multimodal prehabilitation improved preoperative functional capacity, although evidence for reducing postoperative complications was limited.	Indirect clinical	Small number of studies; heterogeneity in nutritional and prehabilitation interventions; limited high-quality evidence.
**Penny et al. (2022)**	Clinical review	Narrative review	Patients with acute and chronic wounds	Not applicable	Not applicable	Not applicable	Not applicable	Wound care / Multiple surgical specialties	Wound healing, nutritional optimisation	Adequate protein intake and correction of vitamin and	Indirect clinical	Narrative review; recommendations derived from heterogeneous

Study	Study category	Design	Population (n)	GLP-1 agent	Indication	Dose / treatment duration	Comparator	Surgical specialty	Outcomes	Key findings	Directness of evidence	Major limitations
										micronutrient deficiencies support collagen synthesis, angiogenesis, immune function and tissue repair throughout wound healing.		evidence with limited prospective surgical studies.

Supplementary Table S2

Search No.	PubMed search strategy	Purpose
**1**	("Glucagon-Like-Peptide-1 Receptor Agonists"[Mesh] OR semaglutide[tiab] OR liraglutide[tiab] OR dulaglutide[tiab] OR exenatide[tiab] OR lixisenatide[tiab] OR tirzepatide[tiab]) AND ("Wound Healing"[Mesh] OR "wound healing"[tiab])	To identify studies evaluating the relationship between GLP-1 receptor agonist therapy and wound healing.
**2**	("Glucagon-Like-Peptide-1 Receptor Agonists"[Mesh] OR semaglutide[tiab] OR liraglutide[tiab] OR dulaglutide[tiab] OR exenatide[tiab] OR lixisenatide[tiab] OR tirzepatide[tiab]) AND ("Perioperative Care"[Mesh] OR perioperative[tiab]) AND	To identify studies investigating the relationship between perioperative nutrition, GLP-1 receptor agonist therapy and surgical outcomes.

Search No.	PubMed search strategy	Purpose
	(nutrition[tiab] OR malnutrition[tiab] OR "nutritional status"[tiab]) AND ("Treatment Outcome"[Mesh] OR "surgical outcome*"[tiab] OR postoperative[tiab])	
**3**	("Glucagon-Like-Peptide-1 Receptor Agonists"[Mesh] OR semaglutide[tiab] OR liraglutide[tiab] OR dulaglutide[tiab] OR exenatide[tiab] OR lixisenatide[tiab] OR tirzepatide[tiab]) AND ("nutritional deficien*"[tiab] OR micronutrient*[tiab] OR "vitamin deficiency"[tiab] OR mineral*[tiab])	To identify studies examining nutritional deficiencies associated with GLP-1 receptor agonist therapy.
**4**	("Glucagon-Like-Peptide-1 Receptor Agonists"[Mesh] OR semaglutide[tiab] OR liraglutide[tiab] OR dulaglutide[tiab] OR exenatide[tiab] OR lixisenatide[tiab] OR tirzepatide[tiab]) AND ("Sarcopenia"[Mesh] OR sarcopenia[tiab])	To identify studies evaluating the relationship between GLP-1 receptor agonist therapy and sarcopenia.
**5**	("Muscle, Skeletal"[Mesh] OR "muscle mass"[tiab] OR "skeletal muscle"[tiab] OR "lean mass"[tiab]) AND ("Wound Healing"[Mesh] OR "wound healing"[tiab])	To identify studies examining the relationship between skeletal muscle mass and surgical wound healing.
**6**	("Glucagon-Like-Peptide-1 Receptor Agonists"[Mesh] OR semaglutide[tiab] OR liraglutide[tiab] OR dulaglutide[tiab] OR exenatide[tiab] OR lixisenatide[tiab] OR tirzepatide[tiab]) AND ("Body Composition"[Mesh] OR "lean mass"[tiab] OR "fat-free mass"[tiab])	To identify studies investigating changes in lean body mass associated with GLP-1 receptor agonist therapy.
**7**	("Glucagon-Like-Peptide-1 Receptor Agonists"[Mesh] OR semaglutide[tiab] OR liraglutide[tiab] OR dulaglutide[tiab] OR exenatide[tiab] OR lixisenatide[tiab] OR tirzepatide[tiab]) AND ("Body Composition"[Mesh] OR "body composition"[tiab])	To identify studies examining body composition changes associated with GLP-1 receptor agonist therapy.
**8**	("Vitamin D Deficiency"[Mesh] OR "vitamin D deficien*"[tiab]) AND ("Treatment Outcome"[Mesh] OR "Surgical Procedures, Operative"[Mesh] OR "surgical outcome*"[tiab] OR postoperative[tiab])	To identify studies examining the relationship between vitamin D deficiency and postoperative outcomes.
